# An extendable optical fibre probe survey meter for naturally occurring radioactive material (NORM) and other weak emitters

**DOI:** 10.1038/s41598-023-39180-9

**Published:** 2023-07-24

**Authors:** H. T. Zubair, D. A. Bradley, M. D. Khairina, Adebiyi Oresegun, A. Basaif, J. Othman, R. Rifiat, F. Hamidi, L. Rahman, A. Ezzadeen, S. A. Ibrahim, S. Mansor, M. Alkhorayef, H. A. Abdul-Rashid

**Affiliations:** 1 Fibre Optics Research Centre, Faculty of Engineering, Multimedia University, Jalan Multimedia, 63100 Cyberjaya, Malaysia; 2 Lumisyns Sdn Bhd, 47600 Subang Jaya, Selangor Malaysia; 3 Centre for Applied Physics and Radiation Technologies, Sunway University, 46150 Petaling Jaya, Malaysia; 4 School of Mathematics and Physics, University of Surrey, Guildford, GU2 7XH UK; 5 Alypz Sdn Bhd, Jalan Industri USJ 1/1, Taman Perindustrian USJ 1, 47600 Subang Jaya, Selangor Malaysia; 6 Department of Radiological Sciences, College of Applied Medical Sciences, King Saud University, P.O Box 10219, 11433 Riyadh, Saudi Arabia

**Keywords:** Electrical and electronic engineering, Optical sensors, Imaging and sensing

## Abstract

We have developed a radioluminescence-based survey meter for use in industries in which there is involvement in naturally occurring radioactive material (NORM), also in support of those needing to detect other weak emitters of radiation. The functionality of the system confronts particular shortcomings of the handheld survey meters that are currently being made use of. The device couples a LYSO:Ce scintillator with a photodetector via a polymer optical fibre waveguide, allowing for "intrinsically safe" inspection within pipework, separators, valves and other such component pieces. The small-diameter optical fibre probe is electrically passive, immune to electromagnetic interference, and chemically inert. The readout circuit is entirely incorporated within a handheld casing housing a silicon photomultiplier (SiPM) detection circuit and a microprocessor circuit connected to an LCD display. A 15 m long flexible PMMA optical fibre waveguide is butt coupled to an ABS plastic probe that retains the LYSO:Ce scintillator. Initial tests have included the use of lab-based mixed gamma-ray sources, measurements being made in concert with a reference conventional GM survey-meter. Characterization, via NORM sources at a decontamination facility, has shown useful sensitivity, covering the dose-rate range 0.10- to 28 µSv h^−1^ (R-squared 0.966), extending to 80 µSv/h as demonstrated in use of a Cs-137 source. The system is shown to provide an effective tool for detection of radioactivity within hard to access locations, in particular for sources emitting at low radiation levels, down to values that approach background.

## Introduction

The need for above-background radiation level determinations are well-documented, not least in regard to the extractive and associated downstream industries, requiring that naturally occurring radioactive material (the so-called NORM-affected media) are handled with due respect to health and safety, waste creation minimisation, and reuse of decommissioned NORM-affected media. Such issues are confronted in for instance the oil and gas (O&G) industry, the minerals benefications industry, scrap metals handling, and materials intended for export. Moreover, within the context of security, there is need for more versatile capability in detection of anthropomorphic sources that for a variety of reasons may only be emitting at weak levels. In both arenas, and in particular for the purposes of clearance, there may be need to make determinations within difficult to access locations, a theme that the present work seeks to confront. Radioactive waste in the upstream O&G business manifests as sludge and scale. The detection of NORM found in the inner parts of various components can be made complex by virtue of the shapes and sizes of the items that are involved (including bends in pipes, often of limited internal diameter), difficult to access locations, and items of lengths of up to some 15 m and more. Safe, regular monitoring of systems and raw materials is essential.

When inspecting pipes for naturally occurring radioactive material (NORM), an issue that the present work will concentrate on, also acting as a platform for other such challenges, determining the presence of radioactivity is a matter of considerable necessity. This may need to be done for emission levels that even approach background, depending on legal requirements, also in respect of the degree of accuracy needed. Long lengths of pipework, separators, boreholes, flow restriction components, cracks, and other tight space localities may need to be checked. Inspection systems should be robust, also with an ability for the systems of detection to work in aqueous and other similar environments, as well as in the presence of explosive gases. In the upstream oil and gas industry, also in other extractive and benefications industries NORM is often found in what are undoubtedly typically hard-to-reach locations. In situations involving bulk quantities of affected materials, working with these can pose a potential risk to workers. Additionally, it can become difficult to dispose of the affected media, recycling of scrap metals requiring validated freedom from detectable radioactivity. Accordingly, measuring radiation at low levels becomes important, not least for the affected industries, including in respect of decommissioning of facilities and the reuse or repurposing of inspected material. Most current commercial radiation measuring devices are not intrinsically safe, are not able to make remote measurements, do not allow for work in aqueous environments and are less effective in making determinations of radioactivity that only result in weak x- or gamma-ray emissions. Even given the potential for stronger beta components (eg. unsupported Pb-210), in such circumstances measurements made externally to pipework or other such shielding may well miss detection of the short-range beta emissions (being of some tens of cm in air alone).

This paper describes development and preliminary results from a new type of survey metre with sensing capability that is based on an extendable optical fibre system. The device comprises of a small, non-electric non-hygroscopic scintillator probe, presently extendable by some 15 m from the hand-held readout unit. Readout is provided by a unit powered by a rechargeable low voltage battery, and can make measurements at relatively long distance, even in narrow and difficult environments. This new device outperforms previous, benchtop versions^1^ in terms of size and radiation detection capability.

## Review of previous work

In previous efforts to construct a real-time, remote operation radiation detector, Jackson et al.^2^ analysed a commercial device consisting of an inorganic scintillation crystal (ZnWO_4_, 3 $\times$ 3 $\times$ 10 mm^3^) linked to a fibre optic cable, the latter directing the luminescence to a CCD camera. The apparatus was calibrated in a restricted radiation environment consisting of beta particles and gamma rays produced by a 2.4 GBq Cs-137 source (predominant gamma emission 662 keV). During the calibration process the dose rates ranged from 0.125 to 10.0 mSv/h. Because of the system high signal-to-noise ratio, the researchers found the device had a minimum output of 0.2 mSv/h. Subsequently, Jackson et al.^3^ reported use of the same system, now with a photomultiplier tube (PMT), tested under aqueous circumstances, with exposures made using a clinical radiotherapy linear accelerator (linac). The latter provided a highly regulated dose rate extending up to 320 Sv/h. The identical technique had previously been proposed by Reddy et al.^4^ in monitoring ambient radiation surrounding the Sellafield Nuclear Reprocessing Plant, ultimately producing an overall three-dimensional source map. In another study, a remote gamma spectroscopy system based on a fibre-optic radiation sensor (FORS) was created for use in nuclear reactors to detect gamma-emitting sources at remote places, identifying radioactive sources using gamma-ray spectra^5^. In each of these circumstances, as also in regard to the work herein, the fibre-optic system allows for investigation of radioactivity at a distance (for example at several metres and more away from the source), ensuring the safety of the workers involved in such inspections.

The amount of scintillation light produced and collected in a FORS is critical for the sensitivity of the detection system. Reduced graphene oxide (RGO)-coated inorganic scintillators in a FORS device for remote gamma-ray spectroscopy has been demonstrated by Kim et al.^6^. The results showed that RGO coating of the cerium doped gadolinium aluminium gallium garnet (GAGG:Ce) improved the counting rate and energy resolution of the FORS. The wavelength of emissions of GAGG:Ce was close to 535 nm, resulting in a higher transmitted light intensity through the RGO membrane. Based on these results, the study suggests that an inorganic scintillator with a high light yield and emission wavelength close to 535 nm would perform well in gamma-ray spectroscopy after RGO coating. Dohler et al.^7^ made note of signal integration time capability for FORS in applications intended to measure for contamination, also in estimating shielding parameters, low-activity sources taking longer to be detected for a source and detector separated by thick concrete material.

Fernandez et al.^8^ studied probes comprising of a CsI (Tl) scintillator of dimensions 1 mm (OD) $\times$ 5 mm (L), coupled to a PMMA optical fibre for use during in situ gamma monitoring around thermonuclear fusion reactors. In the laboratory, exposure to a Co-60 gamma source provided a dose rate of 0.3 mGy/h to 3 Gy/h, revealing saturation at 1 Gy/h. This limitation, attributed to the long response time of CsI (Tl) crystals (0.6–3.4 µs), has lead to consideration of use of a Ce-doped alternative to give a higher dynamic range. In a similar attempt, Kim et al.^9^ monitored radioactive contamination in flowing water pipes, use being made of a Ce-doped GAG (Gd_3_Al_2_Ga_3_O_12_) scintillator, the fixed setup placed externally to the pipe forming a radiation gauge. The study highlighted the effectiveness of the Ce-doped scintillator in measuring radiation levels from a variety of gamma source emissions: 122 keV from a Co-57 source; 662 keV from a Cs-137 source; and 1173 keV and 1332 keV from a Co-60 source. Joo et al.^10^ presented a FORS system that measures gamma radiation from radioactively polluted soil, while Han et al.^11^ have demonstrated gamma spectroscopy with LYSO:Ce (cerium doped lutetium yttrium orthosilicate).

In the studies mentioned thus far, the power source has been predominantly from mains supply, rendering the apparatus unsuitable for field operations. Herein, in particular, we report characterization of a battery-powered scintillation based optical fibre dosimetry system based on the use of a LYSO:Ce scintillator at dose-rates and energies typically provided by NORM sources. The measurements were calibrated against a GM survey meter. In the present system, the radioluminescence (RL) produced by the scintillators propagates within the optical fibre waveguide to a photo-detection system, providing real-time, remote, and intrinsically safe radiation measurements capability. As has been investigated^1^, the particular LYSO:Ce scintillator arrangement provides a workable sensitivity, which although clearly leading to lesser values than that of GM systems, does offer considerable versatility in the practical applications for which it is intended. In particular, LYSO:Ce provides for high density (7.15 g/cm^3^) and high light output (70% of NaI(Tl), 25 photons/keV), leading to improved sensitivity. The additional features of short decay time (~ 40 ns), favourable energy resolution, < 12%, and good radiation hardness point to further improved utility, extending to use in higher dose rate environments.

## Materials and methods

### Apparatus design

The developed system consisted of a battery-powered, handheld unit (designed during the project and manufactured using 3D printing) that accepts an optical fibre input (spectral predominance towards the blue end of the spectrum). The handheld system receives signal from a miniature detector (based on a LYSO:Ce scintillator–custom dimensions of 3 mm $\times$ 20 mm with a TiO_2_ reflective coating from EPIC Crystal Co. Ltd. (Shanghai, China) that helps to confine the generated luminescence and provide low-loss channelling of the luminsecence to the fibre). The scintillator is housed in a light-tight plastic housing, and connected (via butt-coupling) to the far end of a 12.5 m optical fibre channel (SH8001 ESKA™ Super 2.0 × 3.0 mm SH Simplex Cable, Mitsubishi Chemical Co., Tokyo, Japan).Tokyo, Japan) that is mounted on a manually rotatable spool. In the initial state, the optical fiber channel is rolled up and subsequently unwound according to the operator's requirements. This signal is acquired by an off-the-shelf silicon photomultiplier (SiPM) MICRO-FJ-SMTPA-30035-GEVB (evaluation board) developed by SensL Technologies Ltd. (Cork, Ireland), processed by in-house pre-processing and amplification modules capable of handling signals up to 450 MHz and consuming 40 mA on average. The TiO_2_ coating referred to earlier is not expected to contribute at a practical level to the radioluminescence signal, having a relatively low atomic number (Z = 22) and associated low radioluminescence production capability. A microcontroller with 8 bits performed the pulse counting. On a single charge, the entire system's 3.7 V Li-ion rechargeable battery can power it for approximately 33 + hours. The readings were displayed on an embedded digital LCD screen. In addition, the device is equipped with a red LED and a buzzer that activates whenever a predetermined threshold level of radiation is detected.

Development of the current system has sought to address two major challenges:

1. The need for an extendable miniature probe of a range of >10 m, albeit leading to signal strength issues, including:
  - Signal Attenuation. Signal loss along the fibre channel was minimised by using an optical channel instead of traditional copper wire (extending from the silicon photomultiplier) and by selecting an appropriate large core specialty optical fibre made of plastic (instead of the usual glass) to provide flexibility over long distances. Larger core optical fibre having 2 mm diameter opposed to 1 mm core diameter as in Zubair et al.^1^ contributes significantly to light capture and sensitivity improvements;
  - Sensitivity due to small size: The sensitivity of the miniature probe (3 mm diameter, compared to 25 mm diameter in conventional systems) was maximised by applying a titanium oxide reflective paint coating and designing 3D printed structures to support fibre-to-SiPM coupling.
2. The desire for a handheld instrument:
  - Signal Processing circuits require high power, typically supplied by the mains: To circumvent this, a signal processing circuit with low-power components was designed.
  - Handheld devices require batteries with an adequate lifespan: The total system has an ability to operate for over 33 hours, or roughly four days. This was accomplished by designing voltage regulators with the highest possible output efficiency.

**Figure 1:**
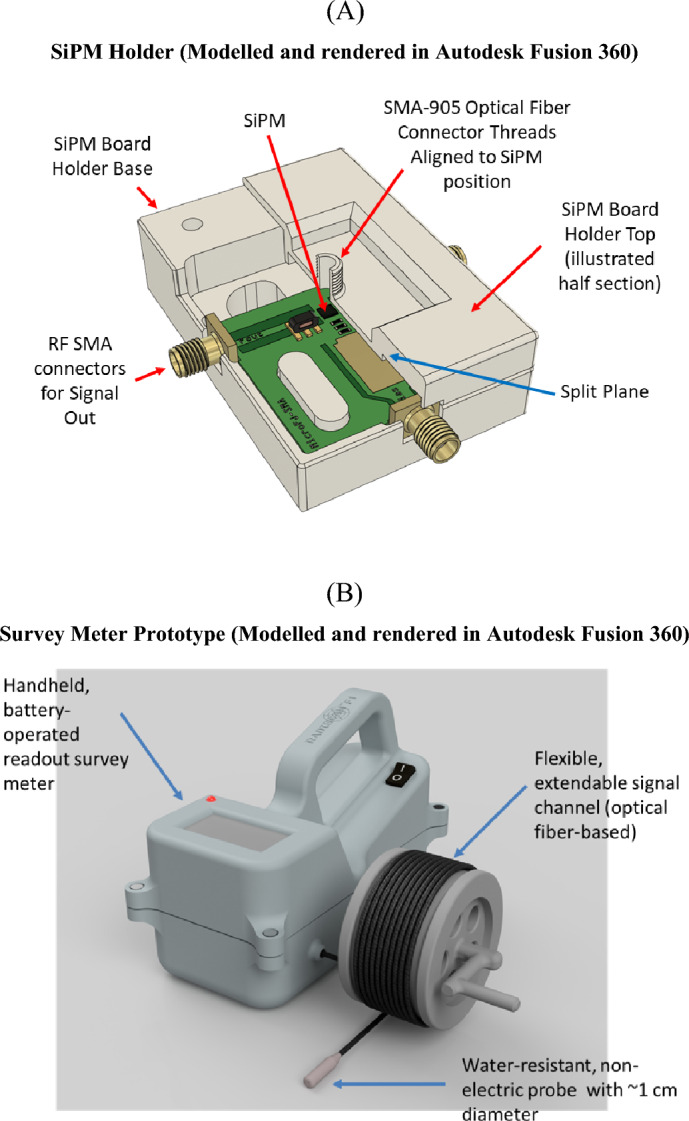
CAD rendering of (**A**) enclosure for SiPM board providing alignment of fibre to photosensor and blocking ambient light (**B**) handheld prototype survey meter with extendable fibre optic probe using Autodesk Fusion 360^12^.

Figure 1A illustrates the stereolithographic (SLA) 3D design of the custom housing for the SiPM board, created using photosensitive black resin. The enclosure has two main functions: first, it inhibits light from reaching the sensor, and second, it keeps the optical fibre connector on top of the sensor to ensure its proper alignment and location. Figure 1B illustrates the CAD rendering (modelled and rendered in Autodesk Fusion 360^12^) of the handheld enclosure, later produced using the SLA procedure (B). The optical fibre channel is mounted on a reel external to the handheld unit, while the SiPM enclosure and the other components are housed inside. The scintillator is butt-coupled and housed inside a specially made plastic ferrule of some 10 mm diameter at the other end of the fibre.

### Signal processing and characterization

The miniature scintillator produces optical emission within the order of nanoseconds (corresponding to a single event of interaction between radiation and scintillator material), captured by the SiPM and converted to photoelectric pulses. These photoelectric pulses are then processed into TTL pulses for a microcontroller to read as digital signals. The registered number of these digital signals in a definite sampling period essentially provides the count rate. As per standard specifications and experimental studies^13^, the LYSO:Ce scintillator has a decay constant of approximately 40 ns as previously mentioned. Ideally, a pulse from this sensor medium should be of shorter duration than 40 ns given that the resultant analogue signal is subject not only to the performance of the photodetector media but also to that of the pre-processing circuitry. In solid-state media for scintillation detection, Dolinsky et al.^14^ have demonstrated favourable response times compared to that of a traditional photomultiplier tube. In the absence of a pulse shaping circuit, the pulse fall-off time of the acquired signal (the time needed in order for a pulse to return to baseline) may be significantly greater than 40 ns.

**Figure 2:**
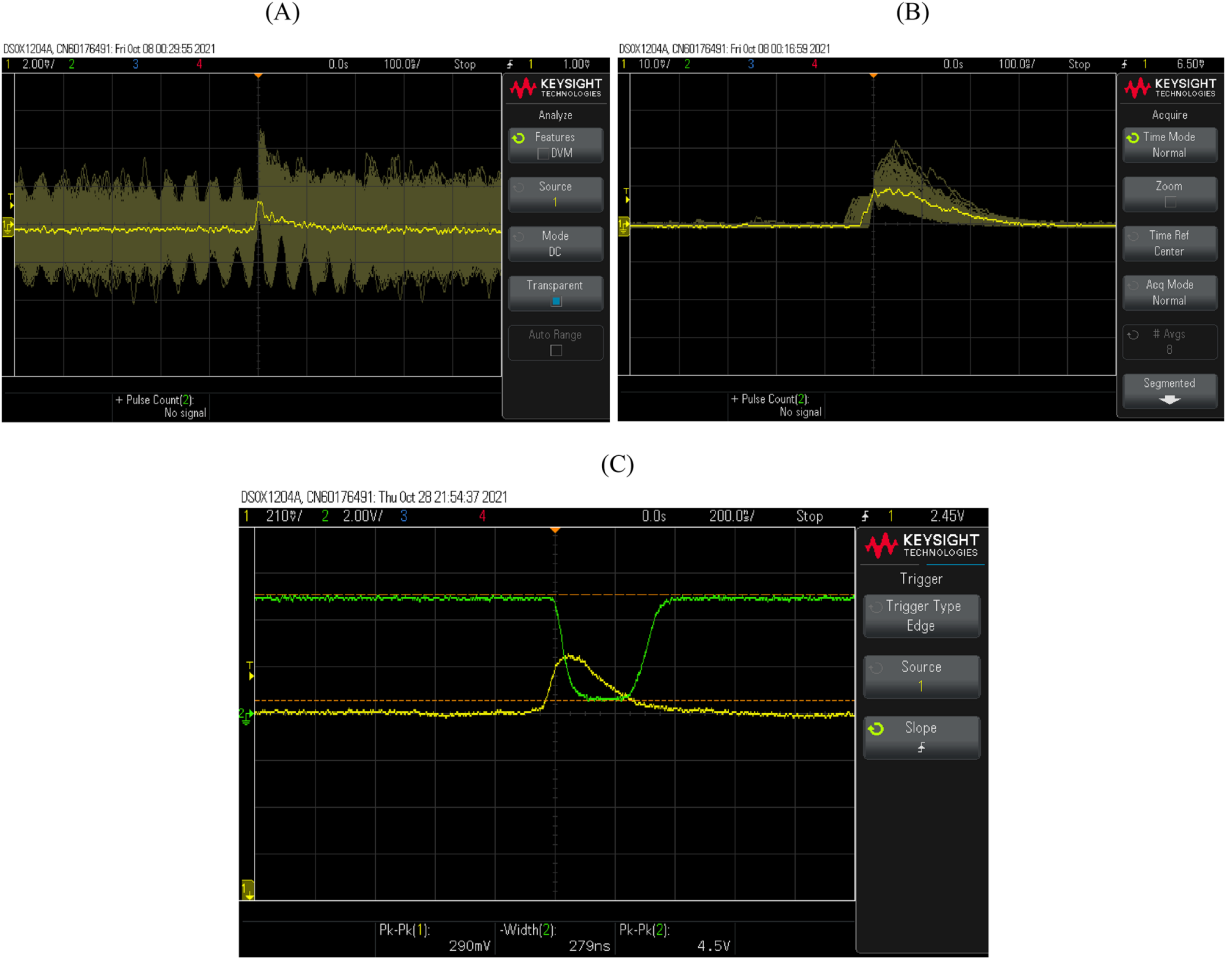
(**A**) Background noise reaching a maximum amplitude of 5 mV, (**B**) raw signals from fibre-coupled scintillator captured at threshold level 6.5 mV, and (**C**) Scintillation signal captured using oscilloscope showing amplified signal (yellow trace) and corresponding comparator output (green trace).

In the current design, the scintillator signal was captured from the "Standard Output" port of the SiPM evaluation board, and the resultant emission decay constant was determined to be 130 ns. When processed through an amplifier, the decay constant was roughly extended to 200 ns. Figure 2A illustrates the response of the detector from a dummy probe (with no scintillator present). This provides the signal levels and count rates of the dark counts, reaching an amplitude of approximately 5 mV. Thus, voltage levels above 5 mV, in this case above 6.5 mV (arbitrarily chosen), were considered to be scintillation signals. Figure 2B presents the signals captured with a threshold set at 6.5 mV. Pulses of several heights were captured, ranging up to 25 mV. The height of these pulses corresponds to the energy of the detected radiation packet. For the purpose of monitoring the dose rate, capturing the time of incidence of the pulses is sufficient.

The raw signals are processed through an in-house developed pre-processing circuit with approximately 15 times gain. Figure 2C illustrates a sample raw-TTL signal pair that was produced as a result of the amplification of the raw signal (represented by the yellow trace), and subsequent processing through a comparator (represented by the green trace). The resultant negative TTL pulse had a width in the order of 200–300 ns. These pulses were then fed to a microcontroller for pulse-counting purposes.

### Experimental setup

Data for this study was gathered for three sample types: Cs-137 (a laboratory source), a mixed gamma source (a laboratory source), and a relatively large dimension NORM source with distributed radioactivity, with both gamma-ray and beta emissions. For each sample, the sources were kept in a fixed position, the probe of the survey meter device under test (DUT) being placed at various separations from the source. A reference Geiger-based survey meter (Radiation Alert® Ranger, S.E. International, Inc., Tennessee, USA) was positioned in the same location as the probe. As shown in Fig. 4A, the Cs-137 laboratory source had an activity of 387.4 kBq on May 1st, 2021, the source providing a dominant gamma-ray emission of 662 keV.

**Figure 3:**
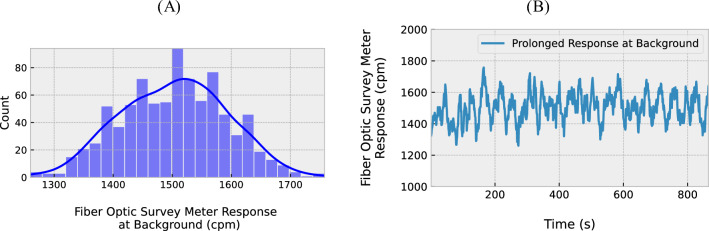
(**A**) Histogram of cpm readings obtained with the DUT, yielding a mean of 1503 cpm and a standard deviation of 89 cpm; (**B**) Plot of background cpm data taken over a prolonged counting period.

**Figure 4:**
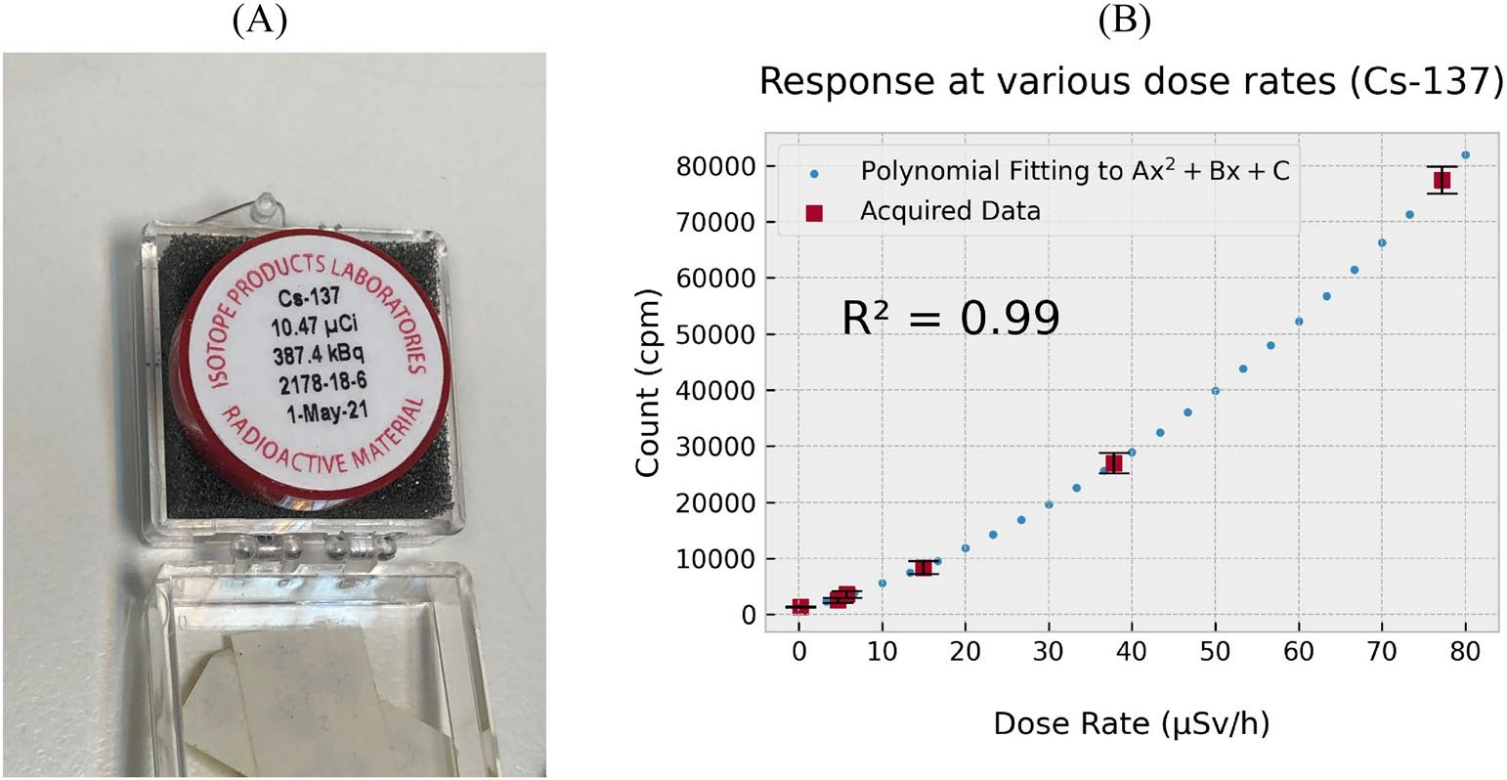
(**A**) Cs-137 laboratory source with activity of 10.47 µCi used as main radiation source for measurements with DUT, and (**B**) Response of DUT in CPM for dose rates between background (0.2 µSv/h) and ~ 80 µSv/h.

**Figure 5:**
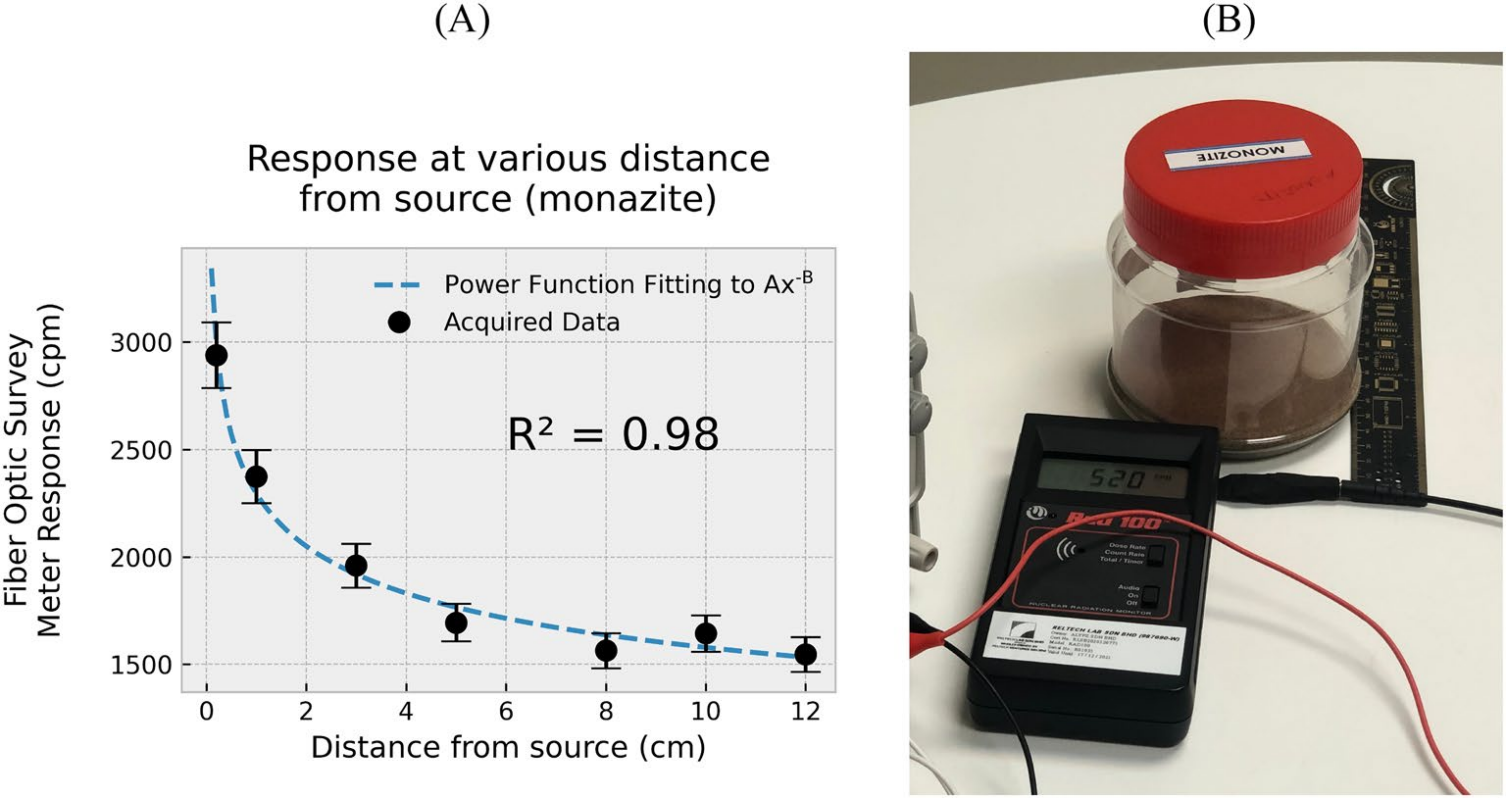
(**A**) Response of DUT in CPM at distances up to 12 cm, and; (**B**) a jar of monazite acting as the source for this part of the study, obtained from laboratory storage and typically used for educational/training purposes.

**Figure 6:**
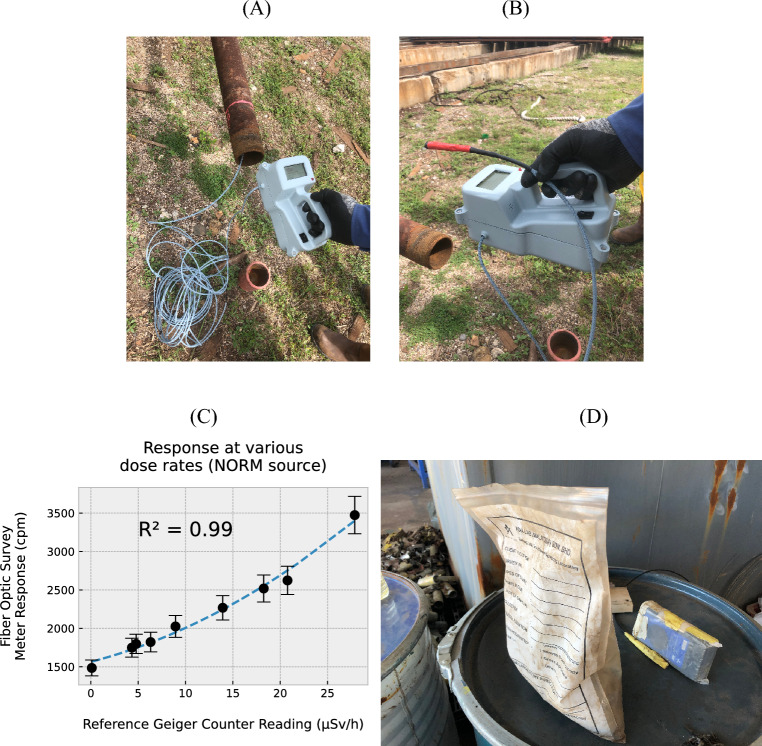
Handheld prototype being operated in an actual NORM environment, with the probe (**A**) positioned inside a tubular; (**B**) illustration of the miniature size of the probe, wrapped in red tape. (**C**) Response of fiber optic survey meter in cpm at various dose rates around a NORM sample pack with a 2nd order polynomial fitting, and; (**D**) Experimental setup in the outdoor environment, with NORM dust (~ 700 g) collected in a pack right after the decontamination process. The probe was placed at various distances from the pack.

**Figure 7:**
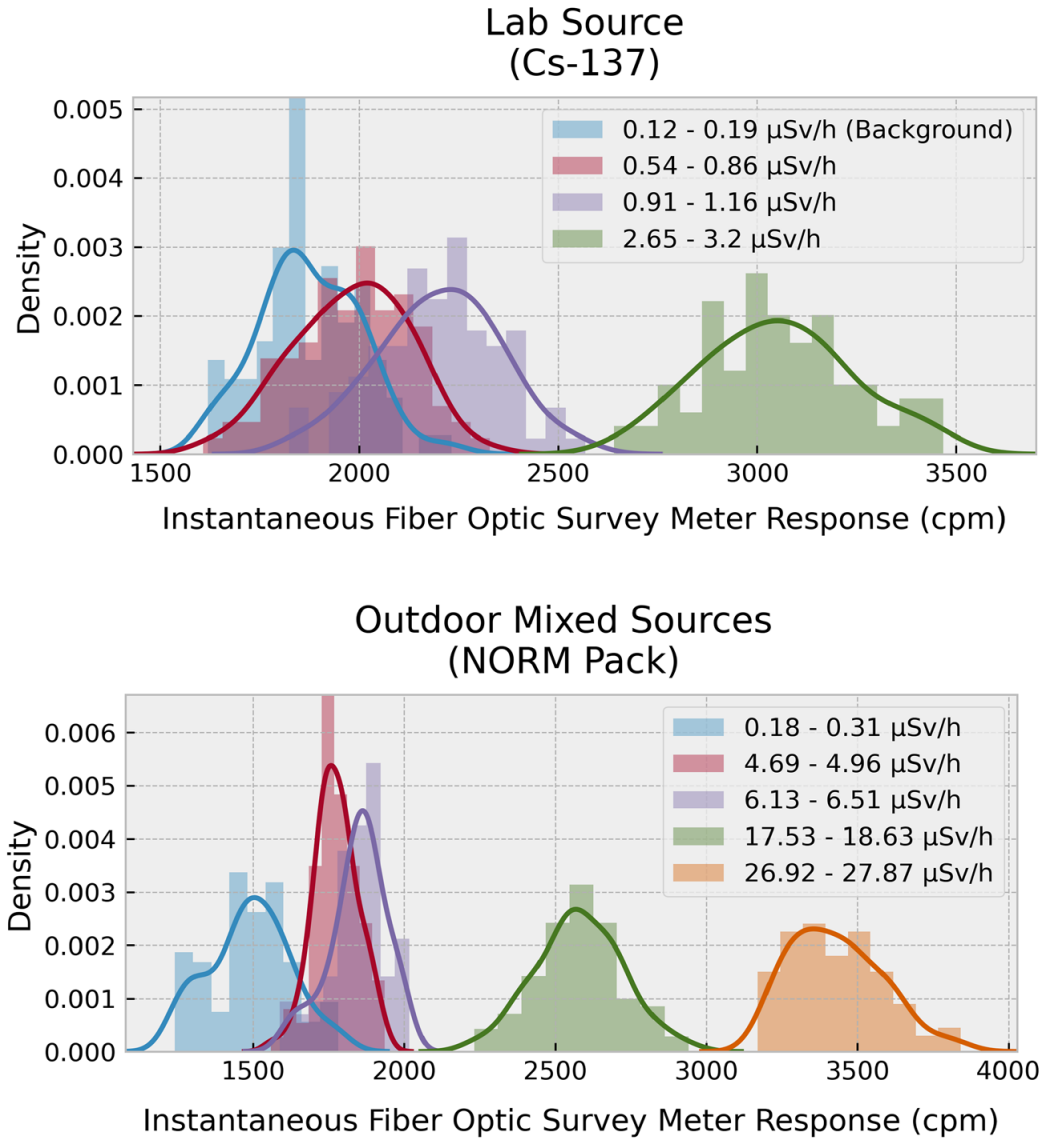
Density plots of radiation counts (cpm) showing distribution of recorded values obtained over a prolonged counting period, with use of the FORS system for laboratory and natural sources and with probe and source in fixed positions relative to each other.

Tests were conducted using a mixture of gamma-ray sources and low activity natural samples (monazite, iron oxide, etc.), all at the laboratory of Alypz Sdn. Bhd. (Malaysia). The same procedure was followed in determining device response as a function of source-DUT separation. Inverse reciprocity between dose-rate and separation, clearly being the expected outcome, is certainly not anticipated to conform to the inverse square law, the latter being determined for a point source and point detector, best approximated at sufficiently great separations. The radionuclide activities in the monazite composite are given in Table 1, these values having been obtained in the Alypz Sdn. Bhd lab using their shielded hyper pure Ge (HPGe) spectroscopy system.

**Table 1:** Gamma analysis for monazite sample (0.36 kg).

Sample	Activity concentration (Bq/g)					
	Ra-226	Pb-214	Bi-214	Ac-228	Pb-212	K-40
Monazite	16.2 ± 1.3	25.5 ± 0.7	23.9 ± 0.4	206.3 ± 3.4	153.0 ± 6.9	non detectable
Minimum Detectable Activity (MDA) within the HPGe shielded cavity	1.650	0.225	0.221	0.152	0.980	0.674

Additional measurements were taken at an O&G tubular NORM decontamination facility located in Terengganu, Malaysia. During readout the probe was moved manually to provide for various separations. The prototype DUT can be seen in Figs. 6 and 7A. The probe was exposed to a pack of NORM (~ 700 g) collected in a post-decontamination exercise within the NORM decontamination facility. Two reference GM survey meters (Radiation Alert® Inspector, S.E. International, Inc., Tennessee, U.S.A. and a Rad 100™, International Medcom, Inc., California, U.S.A.) were also used to record the dose-rate. Measurements were taken at distances of up to 15 cm from the sample, a separation at which the recorded dose-rate begins to approach background radiation levels. The greatest levels were recorded when both the probe and GM survey meter window were in contact with the sample pack.

## Results and discussion

Prior to making measurements on samples, the background level was established. Readings for prolonged exposure to background (at a mean and standard deviation of 1503 ± 89 cpm) are presented in Fig. 3. This corresponds to natural radiation levels from the environment, typically within the range 0.10–0.25 µSv/h. For the same measurement durations as used for the probe and for similar conditions to that found in our laboratory the reference survey meter registered a dose-rate of 0.221 ± 0.030 µSv/h. The standard deviation in observed counts, encompassing natural variation in the background radiation, contributes to the inherent sensitivity of the scintillator.

### Laboratory source (Cs-137)

The DUT was exposed to a Cs-137 source to establish the general sensitivity. The maximum dose-rate captured by the reference survey meter was ~ 80 µSv/h, detected when the device/probe were in contact with the source. Figure 4 (A) shows the 387.4 kBq source (measured some six months prior to the present study) encapsulated within a plastic casing. Observed from Fig. 4B is the greater percentage uncertainty existing at the higher dose-rates. In regard to pulse pile-up effects these are largely unexpected at the low-to moderate count rate levels of interest, anticipated to only become an issue at much higher count-rates of the order of 10^6^ cps, in line with a pulse width of the order of 200–300 ns as mentioned earlier. The noted percentage uncertainty, reducing precision of measurement, is more likely attributable to the construction of the probe, formed in simple butt-coupling of the PMMA light carrying fibre of facial surface corresponding with its 2 mm diameter and the scintillator surface of 3 mm diameter. This mismatch allows a portion of the scintillation to escape entering the PMMA fibre, an effect that can be expected to be proportionally greater at higher dose rates due to greater emission within the scintillator crystal. This remains an area for further improvement in terms of the mechanical design of the coupling arrangement. In its current state, the sensitivity of the probe at room temperature has been estimated to be ~ 450 cpm/µSv/h, corresponding to low dose rates (below 15 µSv/h) and ~ 930 cpm/µSv/h for dose rates at around 15 µSv/h. For practical applications an equation derived from polynomial fitting (of the form Ax^2^ + Bx + C) of the experimental data, or similar such approximations may be used to convert from recorded cpm values to dose rate in µSv/h.

### Laboratory NORM source

In regard to practical scenarios, the utility of the system is foreseen to be in detection of NORM and also other radioactive contaminants confined to narrow, hard to access locations. It can also be used in the search for anthropomorphic materials, down to weak emission levels, seeking out orphaned sources or sources that have been deliberately concealed. To validate system capability, the system was subjected to various weak NORM sources (emitting both gamma-rays and beta particles) as available in the Alypz Sdn Bhd laboratory (from material collected from external sites and retained for training purposes). Figure 5A shows results from this exercise, concerning a jar containing a monazite source. The inverse relationship between count rate and source to probe separation x is seen to follow a form Ax^−B^, the particular power relationship (power of B) fitted to obtain an R-squared value of 0.98. As shown in Fig. 5B, a Rad100 reference survey meter was also placed alongside the probe to allow simultaneous record of the corresponding dose-rate, recording a value of 7.59 µSv/h when in contact with the jar. The mean dose-rates at distances of 12, 10, 8, 5, 3, 1, and 0.2-cm were found to be 1.19, 1.47, 1.98, 2.72, 4.37, 6.44, and 7.59 µSv/h, respectively. As observed in several previous studies^1,7^, at extremely low radiation levels, discrimination between different dose rates requires longer integration time, in the range of tens of seconds. Results for the present arrangement shows effective discrimination at the level of ~ 0.5 µSv/h for data acquired over a period of 30–40 s. A more elaborate discussion regarding responses at low dose rates will be presented in a subsequent subsection.

### Outdoor NORM samples

The functionality of the DUT was investigated in a real NORM-affected scenario (Fig. 6), seeking validation of its utility in the field. Results shown in Fig. 6C refer to the various readings (cpm from the DUT vs. µSv/h from a GM survey meter) obtained for the probe and GM survey meter placed in the close vicinity of collected NORM samples, as shown in Fig. 6D. The probe was first placed as far as 15 cm from a pack of NORM-affected dust, the measured dose-rate approaching background levels. At closer locations, increased dose-rate was recorded. At the closest distance (with contact made with the surface of the NORM pack) the recorded dose-rate was ~ 28 µSv/h, obtaining counts in the range ~ 3400 cpm. Demonstrating the sensitivity of the DUT within an operational environment, a value of 48 cpm/µSv/h was found, also exhibiting the non-linear behaviour as observed in Fig. 4B. Decrease in sensitivity compared to previous settings is noted, attributable to the volume, distribution of radioactivity and beta-particle attenuation within the affected material (spectroscopic evaluations of for instance the lab-based monazite source, made using a hyperpure Ge detector, have shown such samples to have an appreciable Pb-214 content, accordingly with associated beta particle emissions). The situation indicates a need for appropriate calibration protocols in use of optical fibre survey meters, acknowledging the restricted volume of the scintillator, as well as its sensitivity to the different emissions and their characteristic energies.

### Sensitivity and resolution

The sensitivity exhibited by the optical fibre-scintillator system is dependent not only upon the type of source but also upon the volume, shape, and angle made with respect to the probe position and distance from the source^1^. Figure 7 offers a detailed look into the distribution of counts (cpm) observed at specific distances as the probe was placed at various distances from the respective sources. The histograms are labelled according to the minimum and maximum dose-rates recorded using a GM survey meter at particular distances. For exposure from the lab Cs-137 source in the form of a regularly shaped disk, smaller relative to the GM tube but comparable to the dimensions of the scintillator probe (at 1–2 cm), the responses show the sensitivity resolution to be ~ 0.5 µSv/h, taking the median of the recorded counts acquired over 25–40 s. The effective dose rate (measured using GM survey meters) at a particular distance may itself vary between 15 and 20% about the median value (standard deviation). When extended to NORM sources (with samples collected from decontamination facilities and with a random distribution of the material within each pack), the sensitivity drops manyfold. In taking the median of readings over prolonged periods (60–90 s), the effective dose-rate resolution has been estimated to be ~ 2 µSv/h. It is further apparent that in making comparison with the background data of Fig. 3 that the levels shown by the various sources allow discrimination from background, the one exception being for the very lowest activity NORM-affected media, with dose-rates comparable to background.

### Limitations of the study and ethical considerations

The primary limitation of this study has been the restricted access to radiation sources and facilities. The experimental plan, shared prior to experiment dates with the operator, was required to be strictly followed. Accordingly, data collection has been limited to as much as required in satisfying the statistical significance of the results. All experiments have been conducted at sites authorized to hold radiation sources and radioactive materials in the presence of Radiation Officers licensed by the Malaysian Atomic Energy Licensing Board.

## Conclusion

In conclusion, the radiation detector system developed utilizing miniature probes based on LYSO:Ce scintillators demonstrates exceptional performance in measuring natural sources of radiation. This has served to satisfy a particular motivation for the work, namely development of a sensitive system capable of validating the efficiency of clearance of radioactive contamination from the inner surfaces of pipework. In the event of exceptionally low residual levels of activity, potential inhomogeneity in beta/gamma emission could be a secondary consideration that is worthy of future study. The portable device, constructed using 3D printing technology and powered by a battery, offers the capability of providing accurate measurements at a distance of up to 15 m, even within pipework of diameters as narrow as 2 cm. The device is integrated with an audible and visual alert (adjustable to selected dose rate thresholds) and boasts a long battery life of approximately 33+ hours on a single charge. The most significant advantage of this system is its ability to make measurements in hard to access locations for dose rates that range from background levels up to the presently investigated ceiling level of 80 μSv/h with a minimum sensitivity of ~ 450 cpm/µSv/h, a significant improvement over previous arrangements with this detector^1^. This makes it a valuable tool in detection of radioactivity for a variety of scenarios and contexts, security included, and not least in ensuring the safety of individuals within occupational environments.

## Data Availability

The datasets acquired and analysed during the current study are available from the corresponding author on reasonable request.
